# Development of the face-to-face component and recruitment strategy of a primary care digital social intervention for patients with asthma: Qualitative focus groups and interviews with stakeholders

**DOI:** 10.1080/13814788.2024.2407594

**Published:** 2024-09-27

**Authors:** Georgios Dimitrios Karampatakis, Samuel Kimber, Helen E. Wood, Chris J. Griffiths, Stephanie J. C. Taylor, Xiancheng Li, Bill Day, Jonathan Mant, Clare Relton, Jane S. Watson, Viv Marsh, Neil S. Coulson, Anna De Simoni

**Affiliations:** a Wolfson Institute of Population Health, Barts and The London School of Medicine and Dentistry, Asthma UK Centre for Applied Research, Queen Mary University of London, London, UK; b School of Clinical Medicine, University of Cambridge, Cambridge, UK; c School of Business and Management, Queen Mary University of London, London, UK; d Department of Public Health and Primary Care, University of Cambridge, Cambridge, UK; e St George’s Healthcare NHS Trust, London, UK; f Usher Institute, College of Medicine and Veterinary Medicine, The University of Edinburgh, Edinburgh, UK; g Medical School, Nottingham City Hospital, Nottingham, UK

**Keywords:** Primary health care, asthma, peer support, online health community, qualitative research

## Abstract

**Background:**

5.4 million people in the UK have asthma, with one third experiencing suboptimal control, leading to co-morbidities and increased healthcare use. A quarter of people with long-term conditions informally access peer support through online health communities (OHCs). However, integrating online peer support into primary care services to facilitate self-management is a new concept.

**Objectives:**

To develop together with stakeholders the content, delivery, and recruitment strategy of a digital social intervention to promote use of online peer support amongst asthma patients in primary care.

**Methods:**

Data was collected by qualitative, audio-recorded, one-to-one interviews with clinicians, and focus groups with patients with asthma from East London general practices. The topic guide was informed by patient and public involvement work. Data collected was iterative (i.e. new ideas were added to subsequent interviews and focus groups). Verbatim transcripts were uploaded to NVivo12 and thematically analysed.

**Results:**

Twenty patients from several ethnicities participated across five focus groups, and three general practitioners and three practice nurses were interviewed. The study’s outputs included: the intervention’s face-to-face content; content of clinician training; patient-facing leaflets/material; and a survey to recruit eligible patients. An intervention consisting of a structured consultation with a primary care clinician followed by OHC engagement, was developed based on three generated themes: ‘introducing OHCs’, describing how clinicians should introduce OHCs; ‘OHC engagement’, describing factors influencing OHC engagement; and ‘clinician training’.

**Conclusion:**

Findings will assist clinicians in consultations about supporting self-management of patients through OHCs. Future research should evaluate feasibility, effectiveness, and cost-effectiveness of such support.

## KEY MESSAGES

Promoting online peer support in primary care consultations is a novel concept.

The study developed the content of a digital social intervention for patients with asthma.

The findings of the current study will inform primary care clinicians’ consultations on digital social interventions and will be tested in a trial.

## Introduction

In the United Kingdom (UK), 5.4 million people have asthma [1]. About one third experience suboptimal control, resulting in roughly 121,000 Emergency Department attendances, 60,000 hospital admissions, 6.3 million general practice-based consultations, and 1500 deaths, annually [1,2]. Asthma-related costs hover around £1.1 billion/year. Uncontrolled asthma often generates co-morbidities, including mental health issues, which negatively influence quality of life, self-efficacy and management, and ability to function [3,4].

Interventions designed to improve emotional and/or behavioural self-management could enhance asthma control, thus decreasing acquisition of co-morbidities, and mortality [5]. The UK National Health Service, however, has limited capacity to effectively support large numbers of patients in self-managing their long-term conditions (LTCs) [6].

It is estimated that one in four patients with LTCs (about 1.35 million UK patients with asthma) use Online Health Communities (OHCs) to interact with peers (patients with similar health concerns) and access lay advice [7]. Previous literature claims that online peer support could improve health-related outcomes [8], maximise adherence to treatment [9] and assist patients in managing their health and care and developing coping strategies [10,11]. A recent meta-analysis reports that peer support interventions delivered through interactive social media translate to positive health outcomes (e.g. weight loss, reduced resting heart rate, and enhanced overall well-being) [12].

Although online peer support has been available outside formal healthcare in the last 20 years through OHCs (e.g. on Facebook and Reddit), promoting it as part of routine primary care is novel and requires innovative care models [13]. Interactions with clinicians, especially face-to-face, are important in fostering patients’ engagement with digital interventions [14]. We hypothesised that formal integration of online peer support into primary care services could encourage patient self-management and improve asthma control and clinical outcomes at scale, thereby reducing the burden on healthcare systems and providers.

Our previous Patient and Public Involvement (PPI) work with members of the Asthma UK Centre for Applied Research PPI group from across the UK highlighted the importance of social networks in acquiring self-management skills and the likelihood of primary care clinicians, especially nurses, to affect patient behaviours [15]. PPI members envisioned online peer support being promoted by primary care clinicians, via a digital social intervention for patients with asthma. The proposed intervention consisted of two components: a structured, face-to-face consultation with a primary care clinician to introduce norms and values of online peer support and sign patients up to the asthma OHC of the Asthma + Lung UK (ALUK) charity (first component), followed by actual engagement with the OHC (second component).

The ALUK asthma OHC is a well-established platform, moderated by ALUK specialist respiratory nurses, with around 20,000 registered and 2000 active users (i.e. users who regularly login). Previous research on the ALUK asthma OHC revealed the safety and effectiveness of conversations between users and their potential for health-related benefits [10,16].

The current study aimed to develop, together with stakeholders, the precise content and delivery of the face-to-face component of a digital social intervention (i.e. the content of the structured consultation to promote online peer support) for patients with asthma in primary care. Our objectives were developing implementation processes (i.e. how exactly to run the intervention within general practice settings), a recruitment strategy (i.e. how to identify patients eligible for the intervention), and training packages for clinicians delivering the face-to-face component of the intervention.

## Method

### Overview

**Figure 1.**
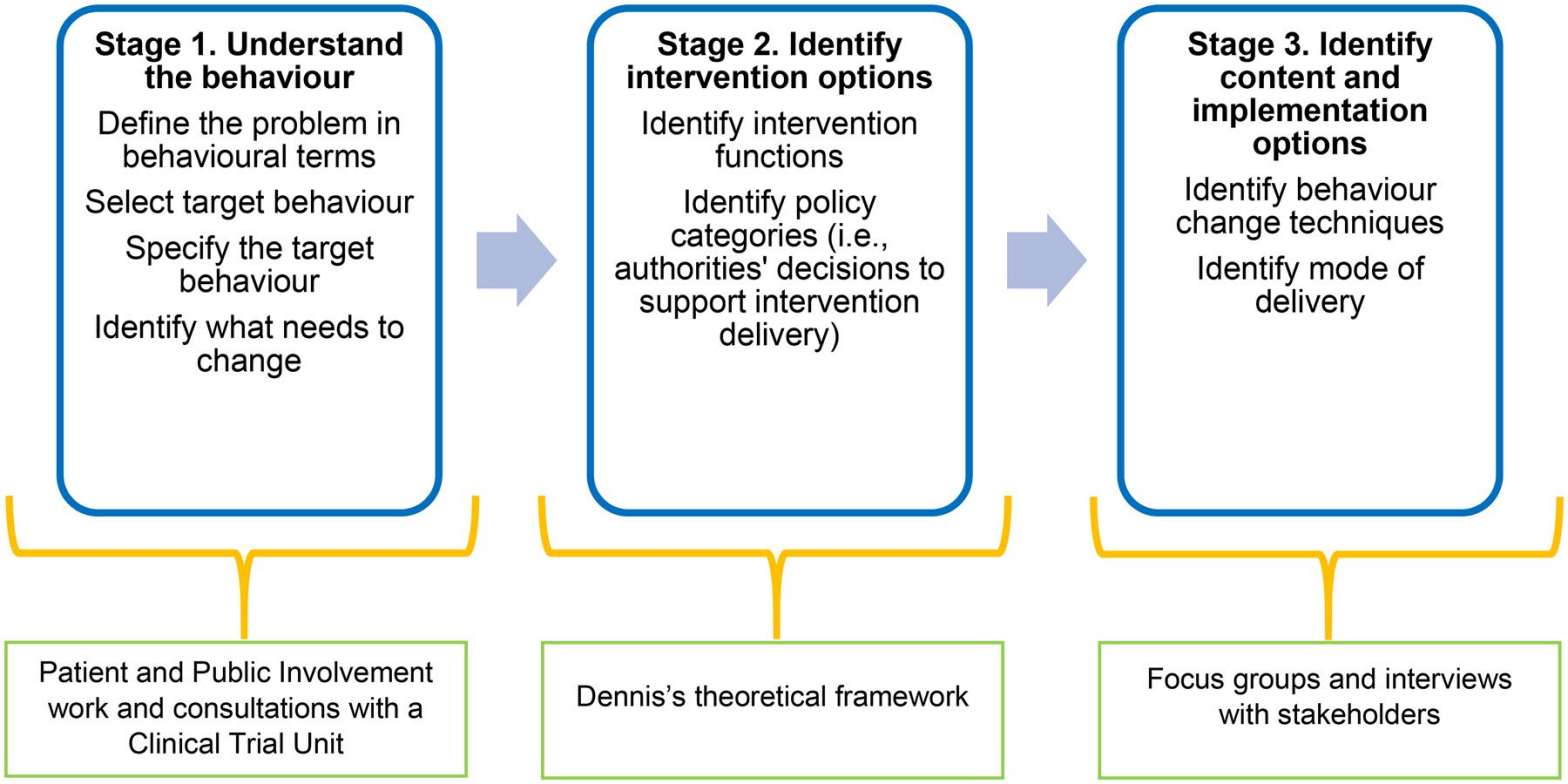
Creaser et al.’s model, as adapted for the needs of our study, employed to develop a digital social intervention for patients with asthma in primary care.

The ‘core elements’ of the digital social intervention were developed according to the Medical Research Council framework for developing complex interventions: by ‘engaging stakeholders’, ‘considering context’ and ‘identifying key uncertainties’ [17]. We followed the practical steps outlined in Creaser et al.’s model for designing health-related interventions [18], which we adapted to the needs of our study (see Figure 1). Creaser et al.’s model encompasses principle elements of the Behaviour Change Wheel (BCW) [19] (see ‘Stage 3′ below for the Behaviour Change Techniques expected to be used in our intervention).

Stage 1 (‘Understand the behaviour’) was based on our previous PPI work (mentioned above) and consultations with staff from a Pragmatic Clinical Trials Unit (PCTU). PPI members conceived the idea of patients with asthma pursuing a behaviour characterised by engagement with an OHC, that could lead to acquisition of self-management skills. For patients to adopt an OHC-engaging behaviour, primary care clinicians need to alter existing work practices by actively encouraging patients to engage with an asthma OHC, within the context of a digital social intervention. Discussions with PCTU staff revealed ‘a survey leading to a trial’ as the best design to evaluate an intervention that only appeals to the subset of the asthma patient population interested in online peer support (this design was successfully employed in a previous trial [20]). The use of a survey as recruitment tool for our intervention enables the identification of patients with uncontrolled asthma, who are interested in online peer support.

We decided to base Stage 2 (‘Identify intervention options’) on Dennis’s theoretical framework, which hypothesises the mechanisms (referred to as ‘effect models’) via which peer support interventions generate possible health outcomes (presented in detail elsewhere [21]). Although guidelines (e.g. as part of clinical templates) might facilitate implementation of a digital social intervention in primary care, the current intervention is yet to be trialled in real time to enable determination of appropriate guidance for clinicians.

Stage 3 (‘Identify content and implementation options’) relies on the findings of focus groups and interviews reported here. From a behavioural point of view, the promotion of online peer support by clinicians entails elements of ‘motivation’, which is among the main ‘targets for intervention’ in the BCW [19]. In terms of specific behaviour change techniques, we anticipate the current intervention to employ ‘prompts’ (clinicians prompting OHC engagement), as well as ‘emotional social support’ and ‘restructures in social environment’ (patients expanding social contacts via OHC engagement). Implementation of the intervention is discussed using Normalisation Process Theory (see Discussion).

### Setting

The study was carried out in three general practices in multi-ethnic, including socioeconomically-deprived, areas of East London. Practices were recruited by the local Clinical Research Network.

### Participants and recruitment

No target sample size was used for this qualitative study. Practice managers invited participation in (a) focus groups by sending text messages to their registered adult asthma patients, excluding those receiving palliative or institutionalised care, and (b) one-to-one interviews by emailing their practice nurses and general practitioners (GPs) involved in asthma care. Texts and emails included a link to an online Participant Information Sheet. Interested patients and clinicians contacted the research team directly.

### Data collection processes

In-person semi-structured focus groups with patients and online one-to-one interviews with clinicians were undertaken between January and April 2023. Before each focus group/interview, participants provided written consent (patients signed a hard copy of the consent form and clinicians filled in an online version of the consent form) and researchers introduced themselves to build rapport with participants. We drafted a single topic guide for the focus groups and interviews based on the findings of our PPI work in Stage 1, which was updated after each focus group/interview to include any new topics arising. Topics included: how online peer support could be promoted by primary care clinicians as part of a digital social intervention; skills needed by clinicians in delivering the intervention; and recruitment of eligible patients via a survey, as suggested in Stage 1 (a draft survey was shared with participants). Focus groups were facilitated by researchers experienced in qualitative research (GDK, HEW and ADS) and a PPI collaborator (BD), who was instrumental in putting participants at ease and ensuring topics were fully understood. Ethnic, language and education differences amongst participants did not generate any major problems with management of the focus groups. Clinicians were interviewed by GDK and ADS. All focus groups and interviews were audio-recorded using digital recorders and the cloud function on Zoom, respectively.

### Data analysis

Audio-recordings were transcribed verbatim by a professional transcriber. The six stages of reflexive thematic analysis by Braun and Clarke were employed [22]. SK and GDK independently coded all transcripts inductively using NVivo12, and regularly debriefed to discuss coding and resolve disagreements, with oversight from ADS. Coded data was sorted into categories, which were re-examined and organised into themes and associated sub-themes. Themes were refined by the whole research team. Reflexivity relied on researchers recognising and putting aside any personal experiences of OHCs. Participants’ feedback on transcripts/findings was not sought.

## Results

In total, 836 text messages were sent to patients. Fifty-six patients and six clinicians expressed interest and were invited to participate, without further selection. Twenty patients attended and participated in five focus groups; all six clinicians were interviewed. Focus groups lasted 1.5–2 h and interviews 15–40 min. Focus groups/interviews ended when participants did not have any further comments to add.

Participating patients were representative of local populations (White, Asian, and Black people of diverse employment status) and asthma severity (asthma severity was not formally assessed but during focus group discussions some described suffering from severe and some from milder forms of asthma). Participants’ demographics are summarised in Table 1.

**Table 1.** Demographics of participants in the focus groups and interviews.

	Gender	Age range (in years)	Ethnicity	Employment status	Years of practice	Previous experience of online health communities
Patients (*n* = 20)	Female (*n* = 13) Male (*n* = 6) Not provided (*n* = 1)	16–19 (*n* = 1) 20–30 (*n* = 5) 31–40 (*n* = 5) 41–50 (*n* = 2) 51–60 (*n* = 5) 61–70 (*n* = 1) 70+ (*n* = 1)	Asian/Asian British (*n* = 6) Black/Black British (*n* = 9) Mixed (*n* = 1) Not provided (*n* = 1) White (*n* = 3)	Full-time work (*n* = 4) Not provided (*n* = 1) Part-time work (*n* = 4) Retired (*n* = 1) Self-employed (*n* = 4) Student (*n* = 2) Unemployed (*n* = 4)	Not applicable	No (*n* = 12) Not provided (*n* = 1) Yes (*n* = 7)
General practitioners (*n* = 3)	Male (*n* = 3)	41–50 (*n* = 1) 51–60 (*n* = 1) 61–70 (*n* = 1)	Asian/Asian British (*n* = 2) White (*n* = 1)	Not asked	15 (*n* = 1) 25 (*n* = 1) 30 (*n* = 1)	No (*n* = 2) Yes (*n* = 1)
Practice nurses (*n* = 3)	Female (*n* = 3)	41–50 (*n* = 2) 51–60 (*n* = 1)	Black/Black British (*n* = 3)	Not asked	8 (*n* = 1) 15 (*n* = 1) 17 (*n* = 1)	No (*n* = 2) Yes (*n* = 1)

Views from participating patients and clinicians on the concepts discussed were complementary and their views have been reported together. Participants’ overall attitudes and enthusiasm towards a digital social intervention in primary care varied.

### Study outputs

#### Format and content of the survey to recruit eligible patients

In the first two focus groups some participants considered paper copies of the survey as helpful for patients with limited digital skills, however most expressed a preference for online completion, emphasising user- and environmental-friendliness. Hence, we decided to distribute the survey exclusively online, a decision that was endorsed in subsequent focus groups and interviews.

The survey was re-drafted iteratively as the focus groups and interviews progressed, notably to change wording, add explanations (e.g. the process of accessing clinical records), and change question order (e.g. mental health questions after asthma-related questions, and the section about interest in receiving the intervention being moved to the end). Participants expressed reluctance about disclosing date of birth, therefore alternative personal information (i.e. home postcode) was requested.

Most participants discounted incentivising survey completion with a payment, highlighting potentially complicating factors (e.g. voucher distribution, possibility of duplicate entries for greater financial gain), while admitting that response rates might be elevated.

The final draft of the survey is in Supplementary material 1.

#### Content of the intervention’s face-to-face component to promote engagement with online peer support

**Figure 2.**
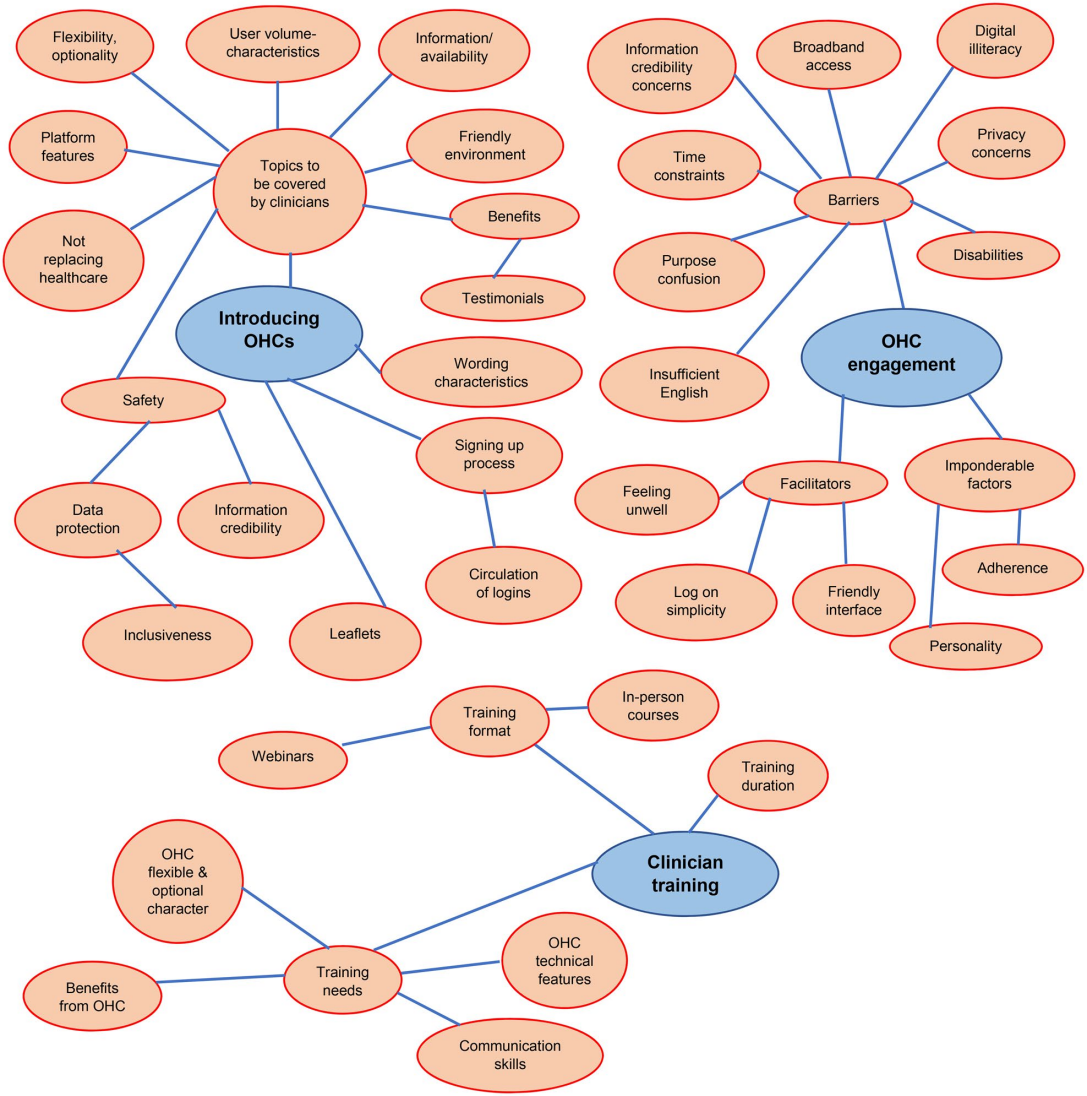
Themes and Sub–themes generated from patients’ and clinicians’ views about content and delivery of a digital social intervention in primary care.

Participants proposed topics that clinicians should cover when introducing online peer support at the consultation, as well as skills they would need, and identified factors that might determine patients’ engagement with the ALUK OHC, as summarised in the three themes generated from focus group/interview data: introducing OHCs; OHC engagement; and clinician training. Figure 2 schematically presents themes and associated sub-themes.

#### Theme 1: Introducing OHCs

Participants emphasised that clinicians should present OHCs as a response to unmet patient needs, with information conveyed positively and succinctly, free of jargon/complicated terminology, and tailored to individual circumstances/needs/interests.

Don’t start with a negative… If you’ve just been told 20 different things [you forget] … ‘Here’s a website that people have similar experiences, and you can get more feedback’, that’s positive… It’s not like ‘if you want more information, you go here’. *(Participant 8, patient)*

Just relate it [introduction to OHCs] to their actual personal diagnosis and situation. How well controlled they are, how well they’re complying with medications… lifestyle… personalise it. *(Participant 26, GP)*

According to participants, clinicians should highlight the large number of OHC users, who are ‘likeminded people’ with lived experience and understanding of asthma.

Let the patient know that they can get advice on how to manage their condition from other people who have dealt with it… a clinician… sometimes… they don’t really get what you’re saying. *(Participant 7, patient)*

Participants suggested that clinicians could introduce the OHC as a resource of immediate and constantly available advice and support, thereby satisfying information and self-care needs.

[Tell patients] it [OHC]’s easier, you don’t need to commute… and they might learn something new… which I might not have picked up on. *(Participant 12, nurse)*

The night-time for me is the worst. I feel like someone is getting on my chest and there’s no-one really to talk to… no access to the doctor… having that online [OHC] access… would put my mind at rest. *(Participant 6, patient)*

We are inundated with requests for consultations with patients who no longer have the ability to self-care. So, something like this [OHC use] will teach them… when they need to titrate up… when to step down. *(Participant 1, GP)*

Participants also felt it was important to emphasise the flexibility in terms of the amount of OHC engagement.

It’s just when it applies to you… that would be a good selling point… You can be there to read and then if you have the information, you can share it as and when. You don’t have to post five times a day or you’re kicked off. *(Participant 11, patient)*

The freedom to express experiences and queries without judgement or time constraint, plus the ease of typing when experiencing asthma symptoms, were perceived as additional points for clinicians to highlight.

[In the OHC] you can express more of what you’re feeling… Whereas in a consultation… it feels extreme, and you feel restricted. *(Participant 14, patient)*

Participants believed that clinicians should highlight the potential health-related benefits from OHC use, possibly by using videos with testimonials from other patients, while clarifying that the OHC would not replace in-person contact with clinicians, viewed as already diminished following the COVID-19 pandemic.

Lastly, it was viewed as imperative for clinicians to refer to the OHC safety, in terms of information credibility (emphasising the presence of nurse moderators, the longevity of the OHC and its oversight by the ALUK charity) and data protection (highlighting the use of anonymous usernames). Reference to data protection was viewed as also implying equality and diversity.

Explain… [that] it’s a trusted website and the type of people [there]… that it’s inclusive… if you could submit stuff anonymously then…. it’s not like it will point out anybody’s race or anything… explain… the safety regulations, GDPR [General Data Protection Regulation]. *(Participant 4, patient)*

Despite some debate, most participants agreed that patients should be signed up to the OHC by clinicians during the consultation, and that clinicians should show patients on a computer screen how to use and navigate the OHC portal.

You ask the patient to do it later… people forget… Having said that, you probably need to give the patient a bit of time to digest all information, but… better to do it [creation of an OHC user account] whilst you’ve got the patient there. *(Participant 17, GP)*

A few participants also proposed the circulation of the OHC link and login details via text messages.

#### Theme 2: OHC engagement

Participants identified multiple factors that might influence patients’ engagement with the OHC, and proposed techniques to maximise it. Limited digital literacy and fluency in English, and lack of Internet access, were viewed as obstacles to engagement.

The use of digital tools… for the elderly… they are not quite good with digital things… Also, some people who don’t work have challenges with the internet… If somebody doesn’t speak English… they will be willing to interact [with OHC peers] … it’s the language barrier which might prevent them from getting the benefit of those groups. *(Participant 2, nurse)*

Some participants recommended translation of the OHC for non-English speakers. A few referred to disabilities, suggesting that volunteer groups could provide relevant assistance, including for patients who are not competent with digital technology.

You’ve got people that can’t read and write properly… they can’t go onto the portal and read it. So, if they could get someone to help them… volunteers that could go around… and say, ‘Well, look, this is what you can do. You might not be able to read this stuff, but you can hear people talking’. *(Participant 19, patient)*

Concerns about privacy, information credibility and time requirements, along with confusion about the reasons for accessing the OHC, were perceived as additional barriers to OHC engagement. On the contrary, feeling unwell was deemed as prompting OHC engagement.

I only engage in things… in time of need. I feel healthy now… so I might not look at it [OHC]. If I start feeling ill – temperature has dropped, start feeling a bit tight chested – that’s probably when I would start to engage… That’s when you remember I’ve got that [OHC]. Let me log on. *(Participant 5, patient)*

Likewise, the simplicity of logging into the OHC (e.g. absence of complicated verification processes) and the presence of a user-friendly and attractive interface were viewed as fostering engagement. To simplify access, participants suggested having the OHC in mobile app format.

Factors that were perceived as difficult to assess included the patient’s personality (i.e. extroverts more likely to interact with other OHC users than introverts) and general adherence to treatment (i.e. adherent patients more likely striving to improve their asthma, including via online peer support).

#### Theme 3: Clinician training

Participants highlighted the need for clinicians to be trained to effectively deliver the intervention and suggested the topics that training packages should cover, including an understanding of the OHC platform, in terms of technical characteristics.

They [clinicians] need to sign up [to the OHC] and be active for a certain amount of time… When I’m trying to sell something to someone… I could sell you on it because I’ve used it myself. *(Participant 11, patient)*

You [clinician] want to know… if I join this community what’s there for me? What’s available? Who is there? What support is available, you just want to know the content of the whole [OHC] thing really. *(Participant 25, nurse)*

Additionally, participants thought training should promote understanding of the potential benefits from OHC engagement for patients and that clinicians needed to acquire communication skills to convey these benefits.

I think it’s [a] motivational interviewing [need]… [might be] lot of training on that sort of [OHC] thing, but that’s going to be the most important thing. *(Participant 26, GP)*

Some participants also mentioned that training packages need to emphasise the flexible and optional nature of the intervention.

[Healthcare] professionals are very tired and exhausted, they do worry about if they say the wrong thing… need to reassure them… this [intervention] is not something… to get scored by… it’s another skill… in their job… it’s not going to put them under pressure… doing extra houses, not getting paid… explaining to them it’s not going to be do or die, it’s a learning process. *(Participant 21, patient)*

Most participating clinicians preferred online training for ease of access and flexibility, while one preferred in-person training to ensure practical and interactive features. All agreed that training should not exceed two hours.

#### Material about the intervention

Participants suggested that patients leave the consultation with a written leaflet, as a reminder of the intervention. The leaflet was designed progressively during the focus groups/interviews and contains a summary of key information about the OHC along with a section to record the patient’s login details (see Supplementary Material 2).

Participants also recommended displaying promotional material about the intervention in practices.

**Figure 3.**
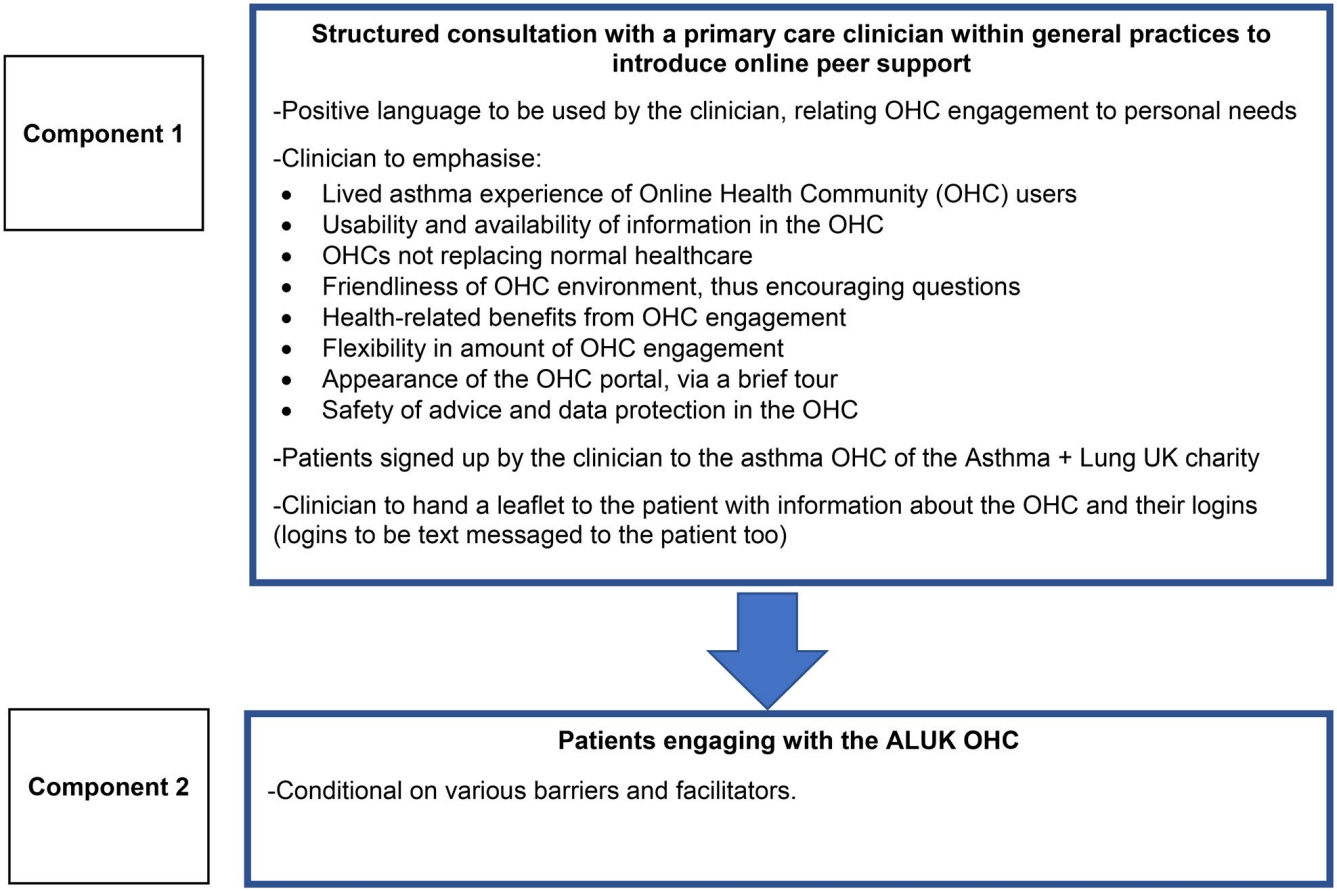
Summary of a digital social intervention in primary care for patients with asthma, as developed together with patients and clinicians.

Figure 3 summarises schematically the intervention developed together with stakeholders.

## Discussion

### Summary

The findings of this study indicate the ‘core elements’ of a digital social intervention in primary care, including how clinicians should introduce online peer support to patients and sign them up to an OHC, practicalities of recruiting eligible patients via a survey, and clinician training. Findings also point to ‘key uncertainties’ with the intervention, namely factors that might influence patient engagement.

### Strengths and limitations

One of the study strengths is the inclusion of various stakeholders’ opinions in developing a digital social intervention in primary care, ensuring exploration of the topic from different perspectives. Participants’ demographics were broad (e.g. clinicians at different career stages, patients from different ethnic and socioeconomic backgrounds), hence findings are representative of diverse priorities and needs. Although these are UK-based findings, insights could be of value for international healthcare systems.

Due to the small sample size and single location, the findings of this study might not be generalisable. It cannot be assumed that results are fully applicable to other long-term conditions, since all participating patients were recruited based on a diagnosis of asthma. In addition, there may be additional safety-related issues for consideration when promoting online peer support in primary care, as not all OHCs are moderated by healthcare professionals. Bringing patients and clinicians together in the same focus groups may have enabled a more thorough discussion. However, this was not attempted since PPI members suggested that patients might not give their full and honest opinions with clinicians present. Not all participants had experience of engagement with OHCs, hence assumptions might be present in the findings. However, our aim was to synthesise a range of possible opinions on an intervention promoting engagement with online peer support, rather than simply capturing experiences of knowledgeable individuals.

### Comparison with existing literature

The intervention developed in this study emphasises the promotion of online peer support to improve emotional and behavioural self-management. Corbin and Strauss report that self-management tasks in LTCs can be differentiated into three categories: behavioural, emotional, and medical management [23]. Previous work with the ALUK asthma OHC showed that users are conscious of the limits of their knowledge, refraining from providing medical advice and instead focusing on behavioural and emotional support [16]. Accordingly, our results highlight that clinicians should emphasise OHCs as a complementary source of information, rather than a surrogate for healthcare.

Interest in engaging with OHCs varied among study participants, with only 30% having previously accessed OHCs (Table 1), consistent with the previous finding that only one in four patients with LTC(s) might be interested in online peer support [7]. Personality-related traits (e.g. inherent extroversion and/or anxiety) might explain discrepancies in OHC interest, something that was also previously described [15].

We found that limited fluency in English, digital literacy, Internet access, and certain disabilities are likely barriers to OHC engagement. Digital social interventions in primary care may widen some health inequalities [24,25]. However, paradoxically, our findings also emphasise the inclusive environment within OHCs. Indeed, previous literature reports that peer support interventions based on interactive social media may be effective for promoting health equity [12,26]. Participants in our study advocated for an exclusively electronic format for the recruiting survey, which emphasises the digital literacy of the majority of the population, regardless of age, ethnic and socioeconomic group. In fact, a recent study revealed that the COVID-19 pandemic has upskilled digital proficiency also in the elderly [27]. Future work to test our intervention (see ‘Implications for research and/or practice’ section below) will consist of effectiveness trials, rather than pragmatic experiments, hence the primary aim will be to explore whether the intervention is effective in a selected population. Nevertheless, equitable access to the intervention will be ensured by recruiting in socioeconomically and ethnically diverse areas.

Normalisation Process Theory is used here to understand how we can move from theory to practice in relation to our intervention. Therefore, despite gaps between research findings and implementation into practice being common, we attempted to interpret our results in the light of Normalisation Process Theory, which conceptualises embedding healthcare interventions into ‘routine work’ (i.e. normalisation) [28]. Normalisation Process Theory’s components include ‘coherence’ (i.e. sense making by parties receiving/delivering the intervention), ‘cognitive participation’ (i.e. commitment by parties), ‘collective action’ (i.e. work by parties for intervention to function), and ‘reflexive monitoring’ (i.e. reflection by parties on intervention). In our intervention, ‘coherence’ relates to patients developing an understanding of OHC norms and values, following consultation with clinicians. ‘Cognitive participation’ relies on clinicians realising and being able to communicate the benefits of engagement with OHCs, through appropriate training. ‘Collective action’ is mirrored in clinicians’ willingness to invest time and effort to effectively introduce online peer support to their patients and in patients’ keenness to engage. ‘Context’ elements potentially influencing ‘collective action’ include workload pressures in general practice settings [29] and lifelong relationships between patients and primary care clinicians [30]. These relationships are being employed to prompt patients’ OHC engagement. To encourage clinicians’ collective action, the intervention is presented as an optional skill for clinicians to employ; relevant training will count towards CPD requirement; and arrangements for intervention-protected time are being made. ‘Reflexive monitoring’ will be collected through interviews with patients and clinicians, as part of future feasibility work and a trial [21], to capture main stakeholders’ views on the intervention.

## Implications for research and/or practice

Research is needed to assess the feasibility and acceptability of the co-developed intervention [21]. Once feasibility is established, a definitive trial will evaluate improvements in health-related outcomes, thus generating evidence-based knowledge about the intervention’s cost-effectiveness.

Our findings shed light on the needs and preferences of a group of patients and clinicians regarding asthma digital social interventions in primary care. These will help clinicians seeking to aid patients with LTCs when patients refer to OHCs during consultations.

## Conclusion

This study developed, together with stakeholders, the content of a digital social intervention for patients with asthma in primary care, including how clinicians should introduce online peer support during the intervention-related consultations, how OHC can be maximised, and what training needs of clinicians should be addressed. The intervention needs to be tested in terms of its feasibility and effectiveness before any widespread adoption. Nevertheless, findings of the current study will enable primary care clinicians to improve their practice by successfully advising on online peer support, thereby fostering the autonomy and agency of their patients. Findings might also be of use to national and international policy attempting to introduce/shape digital social interventions in primary care.

## Supplementary Material

Supplemental Material

Supplemental Material

Supplemental Material

## Data Availability

Data generated and analysed in this study are available from the corresponding author on reasonable request.
