# Supplementary material for: Development of the face-to-face component and recruitment strategy of a primary care digital social intervention for patients with asthma: Qualitative focus groups and interviews with stakeholders
Source: Eur J Gen Pract. 2024 Sep 27;30(1):2407594. doi: 10.1080/13814788.2024.2407594 (PMC11441056; doi:10.1080/13814788.2024.2407594)
Supplement: Supplemental Material [file IGEN_A_2407594_SM9034.pdf]

AD HOC

"A place for you to hear from other people to support you with whatever question you have about your asthma and share your experience"

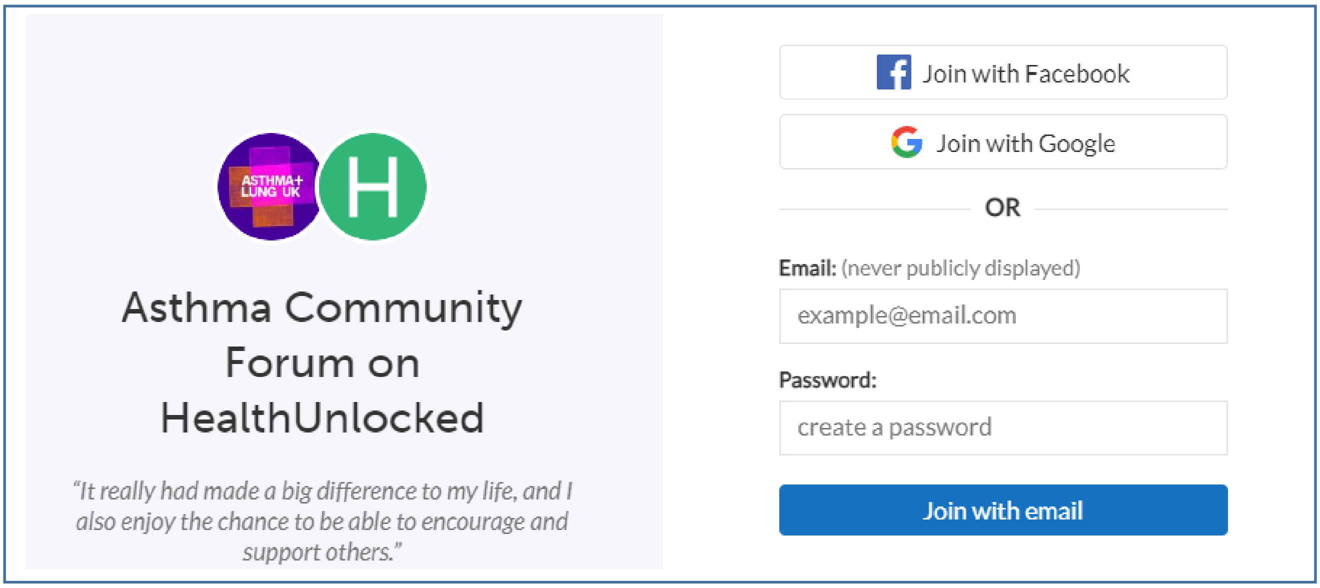

The Asthma Community Forum:

- Is hosted by the charity **Asthma and Lung UK** and moderated by **asthma nurses**;
- Has been running for more than 10 years and has more than 19,000 users (about **1,300 active users**);
- Is **free to use** – you decide if, when and how much you want to engage;
- Uses **your email address** to sign up, but this **will not be shared** with anyone except the researchers in this study.

Your email address is: ……………………………………………………………………
Your username is: …………………………………………………………………………
Your password/password hint is: ……………………………………………………

Thank you so much for taking part!
