# Supplementary material for: Development of the face-to-face component and recruitment strategy of a primary care digital social intervention for patients with asthma: Qualitative focus groups and interviews with stakeholders
Source: Eur J Gen Pract. 2024 Sep 27;30(1):2407594. doi: 10.1080/13814788.2024.2407594 (PMC11441056; doi:10.1080/13814788.2024.2407594)
Supplement: Supplemental Material [file IGEN_A_2407594_SM9031.pdf]

[Page 1: Welcome]

Survey about asthma control and potential interest in an online intervention for improved self-management

Welcome to this survey, the goal of which is to understand the key characteristics of people who are likely to engage with an online health community for asthma, versus those who are not.

The survey includes questions about yourself and your asthma, as well as other long-term health conditions.

The survey should take around 10-15 minutes to complete.

The survey is part of a larger programme of research to design and test an intervention promoting engagement with an online health community for asthma.

If you would like to read more detailed information about this research study, please click on this link to the Patient Information Sheet. **[hyperlink to PDF]**

Thank you for taking part.

[Page 2: Eligibility]

Please only proceed to complete the survey if the following statements are true for you:

1. **I have been invited to take part by receiving a text message from my GP practice** **Yes** ☐ **No** ☐
2. **I have a diagnosis of asthma.** **Yes** ☐ **No** ☐
3. **I am 18 or older.** **Yes** ☐ **No** ☐

I would like to proceed to complete the survey ☐

[must be checked to continue]

[Page 3: Data protection]

About your data

Please note that data collected in this survey will be stored securely by the Pragmatic Clinical Trials Unit at Queen Mary University of London (QMUL).

Information collected will only be accessed by the AD HOC research team.

Any analyses or outputs resulting from this research will only use grouped results. It will not be possible to identify any individual responses, and any identifying information will be removed.

Cookies and personal data stored by your Web browser are not used in this survey.

[Page 4: Instructions]

How to complete this survey

There are three main parts of this survey

1. Questions about your asthma and other health conditions
2. Questions exploring the network of people who might possibly support you when you are unwell
3. Questions about yourself and your potential interest in engaging with an online health community for asthma.

Please read each question carefully and answer all the questions to the best of your ability. There are no right or wrong responses.

At the end of the survey there is a question asking if you would be interested in taking part in future research.

[Page 5. Part 1 questions]

Part 1: About your asthma

Q1. How would you describe your asthma?

☐ Mild
☐ Moderate
☐ Severe

[required, only one answer allowed]

[Page 6. Part 1 questions]

[Asthma Control Test] This section is about your asthma symptoms. For each question, select the answer from the dropdown list that best applies to you.

Q2. In the past *4 weeks,* how much of the time did your *asthma* keep you from getting as much done at work, school or home?

All of the time ☐
Most of the time ☐
Some of the time ☐
A little of the time ☐
None of the time ☐
[dropdown list, required, only one answer allowed]

Q3. During the past *4 weeks,* how often have you had shortness of breath?

More than once a day ☐
Once a day ☐
3 to 6 time a week ☐
Once or twice a week ☐
Not at all ☐
[dropdown list, required, only one answer allowed]

[Page 7. Part 1 questions]

Q4. During the past *4 weeks*, how often did your *asthma* symptoms (wheezing, coughing, shortness of breath, chest tightness or pain) wake you up at night or earlier than usual in the morning?

4 or more nights a week ☐
2 or 3 nights a week ☐
Once a week ☐
Once or twice in the past 4 weeks ☐
Not at all ☐
[dropdown list, required, only one answer allowed]

Q5. During the past *4 weeks,* how often did you have to use your rescue (blue) inhaler or nebuliser medication?

3 or more times a day ☐
1 or 2 times a day ☐
2 or 3 times a week ☐
Once a week or less ☐
Not at all ☐
[dropdown list, required, only one answer allowed]

Q6. How would you rate your *asthma* control over the past *4 weeks?*

Not controlled at all ☐
Poorly controlled ☐
Somewhat controlled ☐
Well controlled ☐
Completely controlled ☐
[dropdown list, required, only one answer allowed]

[Page 8. Part 2 questions]

Part 2: Exploring how you get support with your health

[eHealth Literacy Scale] Q7. This section is about using the Internet for health information. For each statement, select the answer from the dropdown list that best applies to you.

	Strongly disagree	Disagree	Undecided	Agree	Strongly agree
I know what health resources are available on the Internet	☐	☐	☐	☐	☐
I know where to find helpful health resources on the Internet	☐	☐	☐	☐	☐
I know how to find helpful health resources on the Internet	☐	☐	☐	☐	☐
I know how to use the Internet to answer my questions about health	☐	☐	☐	☐	☐
I know how to use the health information I find on the Internet to help me	☐	☐	☐	☐	☐
I have the skills I need to evaluate the health resources I find on the Internet	☐	☐	☐	☐	☐
I can tell high quality health resources from low quality health resources on the Internet	☐	☐	☐	☐	☐
I feel confident in using information from the Internet to make health decisions	☐	☐	☐	☐	☐

[required, only one answer per statement]

[Page 9. Part 2 questions]

Q8. This section is about support with managing your asthma. Who helps you (when needed) with your asthma in relation to the following tasks? For each task, please tick as many boxes as apply to you

	GP	Practice nurse	Pharmacist (in your surgery and/or a in high-street pharmacy)	Other clinician	Family	Best friend	Friends	Asthma support group	Somebody else
**Who help you with taking your medication and attending medical appointments for asthma?**	☐	☐	☐	☐	☐	☐	☐	☐	☐
**Who helps you with adapting your lifestyle and life roles to your asthma?**	☐	☐	☐	☐	☐	☐	☐	☐	☐
**Who helps you with processing emotions that arise from having asthma, for example, when feeling unwell?**	☐	☐	☐	☐	☐	☐	☐	☐	☐
**Who are your most frequent sources of help with your asthma?**	☐	☐	☐	☐	☐	☐	☐	☐	☐

[required, multiple answers allowed per statement]

[Page 10. Part 2 questions]

Q9a. Would you consider looking for advice about your asthma online? Please tick one box.

Yes ☐
No ☐

[required]

Q9b. Please explain your answer [open-ended, text box, not required]

……………………………………………………………………………………………
……………………………………………………………………………………………

Q10a. Would you consider looking for advice online from other people with asthma in a safe and well-moderated online health community (like a forum or Facebook group for people with asthma)?

Yes ☐
No ☐

[required]

Q10b. Please explain your answer [open-ended, text box, not required]

……………………………………………………………………………………………
……………………………………………………………………………………………

[Page 11. Part 2 questions]

Q11a. Would you consider it appropriate for your GP or practice nurse to encourage you to engage in a safe and well-moderated online health community for patients with asthma (a forum for asthma patients who are interested in supporting each other online)?

Yes ☐
No ☐

[required]

Q11b. Please explain your answer [open-ended, text box, not required]

……………………………………………………………………………………………
……………………………………………………………………………………………

[Page 12. Part 3 questions]

Part 3: About your quality of life and mental health

[EQ-5D-5L] This section is about your quality of life. Under each heading, please tick the ONE box that best describes your health TODAY.

Q12a. MOBILITY
I have no problems in walking about ☐
I have slight problems in walking about ☐
I have moderate problems in walking about ☐
I have severe problems in walking about ☐
I am unable to walk about ☐

Q12b. SELF-CARE
I have no problems washing or dressing myself ☐
I have slight problems washing or dressing myself ☐
I have moderate problems washing or dressing myself ☐
I have severe problems washing or dressing myself ☐
I am unable to wash or dress myself ☐

Q12c. USUAL ACTIVITIES (e.g. work, study, housework, family or leisure activities)
I have no problems doing my usual activities ☐
I have slight problems doing my usual activities ☐
I have moderate problems doing my usual activities ☐
I have severe problems doing my usual activities ☐
I am unable to do my usual activities ☐

Q12d. PAIN / DISCOMFORT
I have no pain or discomfort ☐
I have slight pain or discomfort ☐
I have moderate pain or discomfort ☐
I have severe pain or discomfort ☐
I have extreme pain or discomfort ☐

Q12e. ANXIETY / DEPRESSION
I am not anxious or depressed ☐
I am slightly anxious or depressed ☐
I am moderately anxious or depressed ☐
I am severely anxious or depressed ☐
I am extremely anxious or depressed ☐

[required, only one answer per statement]

[Page 13. Part 3 questions]

[EQ-5D-5L continued]

Q13. We would like to know how good or bad your health is TODAY

This scale is numbered from 0 to 100.

- 100 means the best health you can imagine.
- 0 means the worst health you can imagine.

Please click on the scale to indicate how your health is TODAY.

[slider on line can be moved to indicate score, shown in box below slider in backend – as in IBD-BOOST survey. Include markers at intervals of 10, if there is space]

100 - The best health you can imagine

50

0 - The worst health you can imagine

[required]

[Page 14. Part 3 questions]

[PHQ-8] Q14. This section is about symptoms of depression. Over the last 2 weeks, how often have you been bothered by the following problems? For each one, please select the option that best applies to you

	Not at all	Several days	More than half the days	Nearly every day
Little interest or pleasure in doing things	☐	☐	☐	☐
Feeling down, depressed, or hopeless	☐	☐	☐	☐
Trouble falling or staying asleep, or sleeping too much	☐	☐	☐	☐
Feeling tired or having little energy	☐	☐	☐	☐
Poor appetite or overeating	☐	☐	☐	☐
Feeling bad about yourself – or that you are a failure or have let yourself or your family down	☐	☐	☐	☐
Trouble concentrating on things, such as reading the newspaper or watching television	☐	☐	☐	☐
Moving or speaking so slowly that other people could not have noticed. Or the opposite – being so fidgety or restless that you have been moving around a lot more than usual	☐	☐	☐	☐

[not required, only one answer per statement]

[GAD-7] Q15a. This section is about symptoms of anxiety. Over the last 2 weeks, how often have you been bothered by the following problems? For each one, please select the option that best applies to you

	Not at all	Several days	More than half the days	Nearly every day
Feeling nervous, anxious or on edge	☐	☐	☐	☐
Not being able to stop or control worrying	☐	☐	☐	☐
Worrying too much about different things	☐	☐	☐	☐
Trouble relaxing	☐	☐	☐	☐
Being so restless that it is hard to sit still	☐	☐	☐	☐
Becoming easily annoyed or irritable	☐	☐	☐	☐
Feeling afraid as if something awful might happen	☐	☐	☐	☐

Q15b. If you checked off <u>any</u> problems, how <u>difficult</u> have these problems made it for you to do your work, take care of things at home, or get along with other people?

- ☐ Not difficult at all
- ☐ Somewhat difficult
- ☐ Very difficult
- ☐ Extremely Difficult

[not required, only one answer per statement]

Part 4: About you

Q16. How old are you? Please enter your age in years

[number value, limits 18-120, required]

Q17a. Which of the following best describes your race or ethnicity? Please choose one from the dropdown list

White ☐
Black/Black British ☐
Asian/Asian British ☐
Mixed ☐
Other ☐
Prefer not to say ☐
[dropdown list, required, only one answer allowed]

Q17b. Please specify: …………………………………………………
[follow-on question if 'Other' selected, required, free text]

Q18a. How would you describe your gender? Please choose one from the dropdown list

Male (including transgender men) ☐
Female (including transgender women) ☐
Prefer not to say ☐
Prefer to self-describe in another way ☐
(e.g., non-binary, gender-fluid, agender, etc)
[dropdown list, required, only one answer allowed]

Q18b. Please specify: …………………………………………………
[follow-on question if 'Prefer to self-describe in another way' selected, required, free text]

[Page 17. Part 4 questions]

Q19a. What is the highest level of education you have completed? Please choose one from the dropdown list

Primary school ☐
Secondary school up to 16 years ☐
Higher or secondary or further education (A-levels, BTEC, etc.) ☐
College or university ☐
Post-graduate degree ☐
Other ☐
Prefer not to say ☐
[dropdown list, required, only one answer allowed]

Q19b. Please specify: …………………………………………………
[follow-on question if ‘Other’ selected, required, free text]

Q20a. What is your current employment status? Please choose one from the dropdown list

Employed full-time ☐
Employed part-time ☐
Self-employed ☐
Full-time parent/carer ☐
Unemployed, looking for work ☐
Unemployed, not looking for work ☐
Student ☐
Retired ☐
Other ☐
Prefer not to say ☐
[dropdown list, required, only one answer allowed]

Q20b. Please specify: …………………………………………………
[follow-on question if ‘Other’ selected, required, free text]

[Page 18. Part 4 questions]

Q21. How many adults (including you, aged 16 and over) live in your household? Please enter the number

[number value, limits 1-20, required]

Q22. How many children (under the age of 16) live in your household? Please enter the number

[number value, limits 0-20, required]

[Page 19. Part 4 questions]

Before you finish the survey, please enter the following personal details – these will be used only to check that you have not completed the survey more than once and will be stored separately from your survey answers. This information will be stored securely according to the GDPR, only used for the purpose of this study and deleted afterwards.

Q23. First name: ……………………

Q24. Last name: ……………………

Q25. Email address: ………………………………………………………………

Q26. Home postcode: ………………

Q27. GP surgery: ………………….. [ideally dropdown list of enrolled surgeries]

[all questions required]

[Page 20. Health records permission]

My information and next steps of this research programme

We would like to link the answers you have provided in this survey to information about your health from your NHS GP and hospital records, and NHS Digital. We would use this information to collect data about your use of primary and secondary care services because of asthma. If you give your permission, we would use your personal details, provided above (name, email address, home postcode, GP surgery name) to identify your health records. As noted, all data collected will be held securely and will not include identifiable data. This information will be stored separately from your survey answers, only used for the purpose of this study, and deleted afterwards.

Q28. I give permission for my answers to be linked to my health record:

Yes ☐
No ☐

[required]

[Page 21. Interest in future research]

Q29a. Would you be interested in taking part in the next stage of this research, during which you will be signed up with an online health community for asthma? (selecting YES is not a commitment to take part, but we may contact you to tell you more about it)

Yes ☐
No ☐
[required]

Q29b. [If YES] we will contact you using the email address you provided above. If you would also like to be contacted by phone, please provide your mobile phone number [9 digits];

☐☐☐☐☐☐☐☐☐☐☐

[not required]

Q30. Are you already a member of the Asthma + Lung UK online health community or other online asthma community, including Facebook groups for asthma patients?

Yes ☐
No ☐
[required]

[Page 22. Thank you – end page]

Thank you for completing this survey. We very much appreciate you taking the time to contribute to our research study.

If you have said you would be interested in the next stage of our research, we may be in touch with further information in due course.

If you have any questions, or concerns, about this research study, please email us at…
